# Toward Cardiac Regeneration: Combination of Pluripotent Stem Cell-Based Therapies and Bioengineering Strategies

**DOI:** 10.3389/fbioe.2020.00455

**Published:** 2020-05-27

**Authors:** Marta Mazzola, Elisa Di Pasquale

**Affiliations:** ^1^Stem Cell Unit, Humanitas Clinical and Research Center – IRCCS, Rozzano, Italy; ^2^Institute of Genetic and Biomedical Research (IRGB) – UOS of Milan, National Research Council (CNR), Milan, Italy

**Keywords:** cardiac regeneration, induced pluripotent stem cells (iPSCs), bioengineering, cell therapy, 3D-culture system, cardiomyocytes

## Abstract

Cardiovascular diseases represent the major cause of morbidity and mortality worldwide. Multiple studies have been conducted so far in order to develop treatments able to prevent the progression of these pathologies. Despite progress made in the last decade, current therapies are still hampered by poor translation into actual clinical applications. The major drawback of such strategies is represented by the limited regenerative capacity of the cardiac tissue. Indeed, after an ischaemic insult, the formation of fibrotic scar takes place, interfering with mechanical and electrical functions of the heart. Hence, the ability of the heart to recover after ischaemic injury depends on several molecular and cellular pathways, and the imbalance between them results into adverse remodeling, culminating in heart failure. In this complex scenario, a new chapter of regenerative medicine has been opened over the past 20 years with the discovery of induced pluripotent stem cells (iPSCs). These cells share the same characteristic of embryonic stem cells (ESCs), but are generated from patient-specific somatic cells, overcoming the ethical limitations related to ESC use and providing an autologous source of human cells. Similarly to ESCs, iPSCs are able to efficiently differentiate into cardiomyocytes (CMs), and thus hold a real regenerative potential for future clinical applications. However, cell-based therapies are subjected to poor grafting and may cause adverse effects in the failing heart. Thus, over the last years, bioengineering technologies focused their attention on the improvement of both survival and functionality of iPSC-derived CMs. The combination of these two fields of study has burst the development of cell-based three-dimensional (3D) structures and organoids which mimic, more realistically, the *in vivo* cell behavior. Toward the same path, the possibility to directly induce conversion of fibroblasts into CMs has recently emerged as a promising area for *in situ* cardiac regeneration. In this review we provide an up-to-date overview of the latest advancements in the application of pluripotent stem cells and tissue-engineering for therapeutically relevant cardiac regenerative approaches, aiming to highlight outcomes, limitations and future perspectives for their clinical translation.

## Introduction

Cardiovascular diseases represent the major cause of morbidity and mortality worldwide, accounting for 31% of all deaths (Organization WH 2016). Myocardial infarction (MI) is associated with necrosis of the cardiac tissue due to the occlusion of the coronary arteries, a condition that irrevocably diminishes oxygen and nutrient delivery to the heart (Thygesen et al., 2007). While effective therapies, including surgical approaches, are currently used to treat numerous cardiac disorders, such as valvular or artery diseases, available therapeutic treatments for the damaged myocardium are still very limited and poorly effective. Furthermore, after an ischaemic insult, the formation of fibrotic scar takes place, interfering with mechanical and electrical functions of the cardiac tissue (Talman and Ruskoaho, 2016). Indeed, presence of fibrotic scar tissue leads to a reduction of the ejection fraction, due to the altered compliance of the cardiac chambers and to the increased stiffness of the myocardial matrix, thus impairing electrical properties of the heart and consequently the cardiac output (Kim P. et al., 2018). Currently, none of the commonly used therapies are able to remove such fibrotic scar, replacing the lost heart tissue with new functional cardiac cells. Thus, all these pathological conditions result into adverse remodeling of the cardiac muscle and ultimately lead to heart failure (HF), which is associated with poor prognosis in patients who survive a heart attack. The major drawback of all the potential therapeutic strategies for HF is represented by the limited regenerative capacity of the cardiac tissue. In fact, the regenerative potential of the adult human heart is extremely low, with a renewal rate of cardiomyocytes (CMs) of approximately 1% per year (Bergmann et al., 2009). Hence, the ideal approaches to restore the damaged myocardium are to either promote proliferation of resident CMs *in vivo* (Tian et al., 2015; Hashmi and Ahmad, 2019) or to directly provide new CMs for the replacement of necrotic tissue.

In this review, we will particularly focus on those cell replacement therapies based on the use of pluripotent stem cells (PSCs), either embryonic (ESCs – embryonic stem cells) or induced from somatic cells (iPSCs – induced pluripotent stem cells). Indeed, over the last 15 years, the discovery of iPSCs has opened a new chapter in the field of regenerative medicine for the treatment of degenerative disorders, including HF (Takahashi and Yamanaka, 2006). Similarly to ESCs, iPSCs possess the unique ability to differentiate into all cell types of the body, and therefore are emerging as a promising source of cells for regenerative medicine purposes. Furthermore, being generated from patients’ somatic cells, iPSCs overcome the ethical limitations related to the use ESC derivatives and those related to immunological issues, providing an autologous source of human cells (Gonzales and Pedrazzini, 2009).

Pluripotent stem cell-based therapy has already demonstrated some beneficial effects, including the promotion of cell angiogenesis, increased vascularization, attenuation of cardiac cells apoptosis and the reduction of myocardial fibrosis (Gong et al., 2013; Sánchez et al., 2013; Sun et al., 2014; Traverse et al., 2014).

However, despite the initial enthusiasm generated this evidence, several issues have emerged over the years, limiting full application of PSCs to cell replacement-based therapeutic approaches for treatment of HF. Indeed, the low level of maturity of CMs generated from PSCs (PSC-CMs) and the related arrhythmogenic potential *in vivo*, together with the poor grafting of these cells into the heart after transplantation, are still major unsolved problems linked to the use of PSC-CMs for cell replacement therapy (Liu et al., 2018; Muller et al., 2018). Over the last years, research in the cardiovascular field has moved toward the implementation of bioengineering technologies to ameliorate both, survival and functionality of human CMs derived from iPSCs (iPSC-CMs). The combination between the bioengineering and stem cell biology fields has brought to light different technologies able to reproduce the three-dimensional (3D) environment of the heart, mimicking the real structure and function of cardiac tissue. In the same direction, the possibility to directly convert fibroblasts into CMs using microRNAs or small molecules, have recently emerged as a promising area for *in situ* cardiac regeneration.

This review aims to provide an updated overview on cell-based therapies and tissue-engineering, elucidating current applications and limitations, with a focus on future perspectives for their actual application in the clinics.

## Historical View on Pluripotent Stem Cells: From Discovery to Application to Human Diseases

There are two different types of pluripotent stem cells (PSCs): embryonic stem cells (ESCs) and induced pluripotent stem cells (iPSCs). ESCs were first isolated in 1981 (Evans and Kaufman, 1981; Martin, 1981) from the inner cell mass of a mouse blastocyst; more than a decade later, in 1998, Thomson et al. (1998) successfully derived ESC lines from humans. Both, mouse and human ESCs have shown the ability to spontaneously differentiate into various cell types when cultured in absence of the factors required to maintain pluripotency (i.e., LIF – leukemia inhibitory factor or bFGF – basic fibroblast growth factor). By using different protocols, researchers have been able to obtain several different cell types, including CMs and endothelial cells (ECs), and to demonstrate their potential therapeutic value *in vivo* in preclinical models of HF (Laflamme et al., 2007). However, use of ESCs for cell therapy applications in human is highly limited due to their potential immunogenicity and the ethical issues related to human embryo manipulation.

**FIGURE 1:**
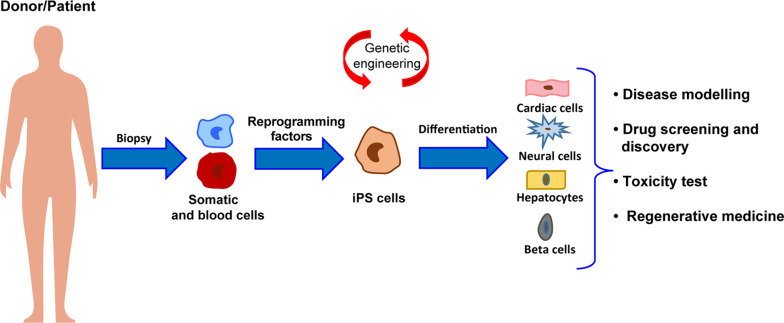
Schematic representation of the workflow for iPSC generation and differentiation from patients somatic cells and major applications to human health.

In 2006, the groundbreaking discovery of Takahashi and Yamanaka (2006) – the possibility to reprogram an adult somatic cell back to a pluripotent state – has given new hope to the regenerative medicine field, potentially allowing to overcome immunogenic and ethical limitations of ESCs to cell therapy. Reprogramming of somatic to pluripotent cells, namely induced pluripotent stem cells (iPSCs), has been first described in mouse fibroblasts by the retrovirus-mediated transduction of four transcription factors (c-Myc, Oct3/4, SOX2, and KLf4), now commonly referred as “pluripotency factors” (Takahashi and Yamanaka, 2006). A year later, iPSCs were also obtained from human cells (Takahashi et al., 2007; Yu et al., 2007). iPSCs share their main features with ESCs, showing the ability of self-renewal and the capacity to differentiate into derivatives of the three germ layers. Indeed, genome-wide analyses revealed that, with few exceptions, human iPSCs and ESCs are mostly similar from the transcriptional and epigenetic point of view (Yu et al., 2007). However, on this regard, an advantage of human iPSCs over their embryonic counterpart resides in their genetic background, which is identical to the donor cell they are derived from Figure 1. Therefore, use of iPSCs promptly became the strategy of choice for studying human diseases *in vitro* (Park et al., 2008; Moretti et al., 2010; De Santis et al., 2017; Salvarani et al., 2019), and their application to drug screening (Yoshida and Yamanaka, 2010; Elitt et al., 2018; Farkhondeh et al., 2019) and toxicity testing is exponentially growing (Egashira et al., 2011; Luz and Tokar, 2018). Indeed, patient-specific lines have been developed in many laboratories worldwide and employed to investigate both functional and molecular phenotypes of a specific disease (Park et al., 2008; Moretti et al., 2010; Sun et al., 2012; Gu et al., 2017; Salvarani et al., 2019). Of note, iPSCs have been successfully differentiated into functional cardiovascular-relevant cells, including cardiomyocytes (Batalov and Feinberg, 2015), endothelial (Orlova et al., 2014), and smooth muscle cells (Xie et al., 2008), and thus have brought new hope to cure cardiac diseases, in particular in the field of cardiovascular regeneration. Additionally, recent advancements in genome-editing strategies by CRISPR/Cas9 technology have greatly expanded the potential of investigation of iPSC-based models, allowing the generation of isogenic iPSC lines, in which a desired gene mutation is either introduced or corrected, and the development of model systems that can be used for modulating endogenous genes expression and for genetic screening of genes function (Hockemeyer and Jaenisch, 2016).

**FIGURE 2:**
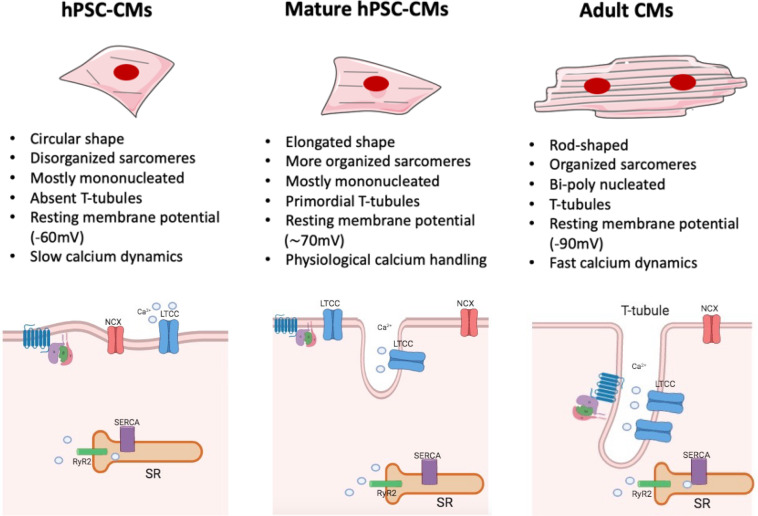
Maturation features of hPSC-CMs. The figure shows a schematic representation of structural and functional features of hPSC-CMs **(left)**, mature hPSC-CMs **(central)**, and adult human CMs **(right)**. Some of the most relevant changes are listed (For a complete view, please refer to Karbassi et al., 2020). hiPSC-CMs **(left)** are mostly mononucleated, showing circular shape and a disorganized sarcomeres’ structure compared to their adult counterpart **(right)**. The absence of T-tubules leads to the lack of organization of LTCC channel, which are not associated with the RyR complexes; such architecture generates slower calcium dynamics and abnormal excitation contraction coupling. Mature hiPSC-CMs **(center)** are still mostly mononucleated but start to exhibit some characteristic tipical of adult CMs: they show an elongated shape together with a more organized sarcomere structure and primordial T-tubule, which lead to physiological calcium handling. However, despite the increased level of maturation reached by mature hPSC-CMs, these cells still lack to achieve a complete functional maturation, as in the adult cardiac tissue. NCX, sodium–calcium exchanger; RYR2, ryanodine receptor 2; SR, sarcoplasmic/endoplasmic reticulum; LTCC, L-type calcium channel.

However, the immature phenotype shown by cells differentiated from human PSCs still significantly limits their full applicability. Indeed, compared to adult human CMs, those derived from human PSCs display a fetal-like sarcomeric organization (i.e., absence of T-tubules and intercalated disks; shorter sarcomeres) and metabolism (i.e., less mitochondria; glycolysis-based energy production). Furthermore, hPSC-CMs exhibit distinct electrophysiological characteristics (diverse repertoire of ion channel densities and longer action potentials) and reduced force of contraction, due to an abnormal excitation-contraction coupling and calcium handling. In fact, in hPSC-CMs diverse components of calcium dynamic processes are missing, such as transverse tubules (T-tubules), invagination of the membrane in which channels and receptors key for impulse propagation and contraction are located (Figure 2). A comprehensive review on recent advances in the field of cardiomyocytes maturation and their implications for regenerative medicine is given by Karbassi et al. (2020).

In the context of tissue repair, this immature state may lead to lethal arrhythmia after hPSC-CMs transplantation (Chong and Murry, 2014). Thus, strategies aiming to differentiate these cells toward a more mature adult-like phenotype would significantly improve their therapeutic value (Yang et al., 2014; Scuderi and Butcher, 2017; Machiraju and Greenway, 2019; Karbassi et al., 2020). Many approaches have been proposed so far, spanning from the maintenance in culture for an extended period of time (Sartiani et al., 2007; Lundy et al., 2013), to the use of biochemical (i.e., growth factors and co-culture strategies) (Yoshida et al., 2018; Selvaraj et al., 2019) electrical stimuli (Nunes et al., 2013; Hirt et al., 2014) and mechanical load (Leychenko et al., 2011; Haggart et al., 2014); among them, those combining 3D culture and bioengineering methods have been the most effective (Tiburcy et al., 2017; Ronaldson-Bouchard et al., 2018; Ulmer et al., 2018).

## Cells-Based Therapy for Myocardial Infarction

The potential to repair cardiac tissue by cell grafting has attracted the attention of the field, due to the scarce capacity of CMs to proliferate and replace the damaged tissue. The dispute over the existence of cardiac progenitors in the adult heart is still unsolved; on the same trail, the ability of immature cardiac progenitor cells to engraft with pre-existing CMs *in vivo* has not been irrefutably determined (Witman and Sahara, 2018). Due to their unique capacity to produce functional CMs, both, ESCs and iPSCs, are among the most promising sources for cardiac regenerative medicine application. The first approach that employed CMs derived from PSCs for cell replacement, was simply based on the injection of the cells directly into the infarcted area of the myocardium. Since 2007, iPSC-CMs were employed in numerous studies for inducing cardiac regeneration after myocardial injury in diverse animal models, including rats, mice and pigs (Caspi et al., 2007; Laflamme et al., 2007; van Laake et al., 2007; Nelson et al., 2009; Ye et al., 2014; Liu et al., 2018). Zwi-Dantsis et al. (2013) used CMs differentiated from human iPSCs derived from patients with HF and demonstrated that, when transplanted in rats hearts, these cells were able to survive and to engraft. More recently, Rojas et al. (2017) developed a genetic-based protocol to maximize purity of CMs obtained from iPSCs differentiation, showing an improvement of the engraftment within the myocardium of mice that have been subjected to left anterior descendant (LAD) coronary artery ligation; in this study the authors also reported a contribution of the transplanted cells to the recovery of the left ventricular function. In another recent study, Zhu W. et al. (2018) investigated whether overexpressing the cell cycle activator cyclin D2 in human iPSC-CMs could improve the graft size through the induction of proliferation of the transplanted cells. The obtained results supported the authors’ hypothesis, demonstrating that cell cycle progression induced by cyclin D2 overexpression was accompanied by a significant increase in heart repair.

Despite the encouraging results from these and other *in vivo* studies on the potential beneficial effects of iPSC-based cell therapy for HF, preclinical experiments in non-human primate models, using CMs derived from human iPSCs, have given controversial results (Chong et al., 2014; Zhu K. et al., 2018). Chong et al. (2014) showed that the injection of human ESC-CMs into failing myocardium of non-human primates was able to promote heart regeneration; however, they also reported the concomitant occurrence of ectopic arrhythmic events, most likely elicited by the immature phenotype of the injected cells. Indeed, as already mentioned above, CMs differentiated from human PSCs differ significantly from their adult counterpart, while possessing structural, molecular, metabolic and functional characteristics comparable to CMs at the fetal stage. Indeed, human PSC-derived CMs possess a spontaneous contractile activity (probably the main reason behind their arrhythmogenicity) and show a poorly organized contractile machinery due to, among others, the reduced sarcomere length and their immature organization, the low expression of beta adrenergic receptors, ion channels and calcium binding proteins. Hence all of these factors together make the electrical and mechanical integration of these cells within the adult cardiac tissue extremely difficult, and significantly increase the potential incidence of arrhythmic events. As a matter of fact, in order to be effective, the newly injected CMs should rapidly achieve the functional features of the host cells, including all of the components of the excitation–contraction coupling machinery, such as gap junctions and transverse T-tubules, which are crucial for the proper functioning of the heart. On this regard, in the trial MAGIC, Menasche et al. (2008) evaluated the effect of transplantation of autologous skeletal myoblasts into the heart after myocardial infarction and found that these cells failed to improve cardiac function, most likely because of their poor coupling with the host myocardial cells.

Another important issue to be considered to properly evaluate the success of heart cell transplantation, is represented by the time-points at which functional measurements are carried out; indeed, the outcomes of these analyses may significantly differ depending on whether they are carried out early (i.e., 4 weeks) or at later stages (i.e., 12 weeks) post intervention (Laflamme et al., 2007; van Laake et al., 2008). In particular, using echocardiography or magnetic resonance imaging, van Laake et al. (2008) reported a significant increase of cardiac functionality at 4 weeks after cell injection. However, such functional improvements faded 12 weeks after MI, despite the initial engraftment, indicating the need to perform analyses at later endpoints. Another variable that may impact the efficacy of cell replacement therapy approaches lies in the number of transplanted cells: the definition of the ideal amount of cells to be transplanted in order to improve heart function and repair without induction of side effects is indeed a critical step, on which a common view is still missing. An additional obstacle to cell-based therapy is represented by the extremely low rate of stem cell retention, a condition that influences the engraftment into the host cardiac tissue (Lemcke et al., 2018; Park et al., 2019). Thus, researchers have started to abandon those approaches based on the injection of isolated cells, moving toward the development of strategies that may optimize coupling with the host. The first attempts in this direction regarded the use of cell-patches (formed by fibrin gel or collagen), that showed an improvement of cardiac function (Hamdi et al., 2009; Vallée et al., 2012; Wendel et al., 2015). Menasché et al. (2015) reported the first clinical application of cardiac patches made using human ESC-derived cardiac progenitor cells in a patient suffering from severe ischemic left ventricle dysfunction, demonstrating the possibility to use cells combined with a tissue-engineered construct for cardiac repair. In detail, ESC-derived cardiac progenitor cells were embedded into a fibrin scaffold, which was surgically delivered into the failing cardiac tissue of the patient suffering from severe heart failure. Although results from this study were encouraging, a greater number of clinical cases is mandatory in order to establish a standard and safe therapy applicable to a higher number of patients. Another approach, proposed by Park et al. (2019), showed a substantial increase in terms of cell retention using a patch loaded with a combination of human iPSC-CMs and mesenchymal stem cell in rat models of MI. The patch was generated using a bioink formed by a mixture containing hiPSC-CMs and porcine heart-derived decellularized extracellular matrix in presence of 0.02% (w/v) of vitamin B2, that was then printed in a disk-shaped polycaprolactone.

However, despite the fact that many different types of stem cells and derivatives have been proposed as valuable candidates for regeneration of the myocardium, a general consensus on which cell type should be considered the gold standard for cell replacement therapy of the heart, remains elusive. This issue mostly arises from the complex structure of the cardiac muscle, that is formed by different types of cells (mainly cardiomyocytes, fibroblasts and endothelial cells), all contributing to the proper functionality of the heart. By now, the majority of preclinical studies have been conducted in two-dimensional culture systems, a condition that does not take into account the multiple interactions occurring in a three-dimensional structure, like the heart: this is among the major issues still limiting the application of cell therapy approaches into the clinics. Thus, in the last years, many studies have directed their efforts to identify the optimal structural and environmental conditions for the development of a three-dimensional (3D) structure able to functionally resemble the cardiac tissue and suitable for heart transplantation. Thanks to the combination of bioengineering methods and the advances in the cardiovascular stem cell biology field, in particular in the protocols for generating CMs from PSCs, significant progress has been made in the development of 3D-cardiac tissue-like structures and their application to cardiac regeneration. The intent of this review is to provide an up-to-date overview of the available technologies and the recent advancements in the field, particularly in relation to their potential applicability for clinical purposes.

## 3D-Culture Systems and Cardiac Regeneration

The function of each organ of the human body is determined by both cellular and non-cellular components. Ideally, a cell culture system that aims at reproducing an organ *in vitro* should be able to replicate the complex interactions occurring between extracellular environment, cells and specialized tissues within the organ itself, as well as their specific functions. In particular, the cardiac tissue is composed by contractile and non-contractile cells (i.e., cardiomyocytes, fibroblasts, and blood vessel cells), that are organized as a complex 3D structure. The tightly regulated interaction among these diverse cell types plays a central role in the heart’s function. Although 2D-cell cultures have been – and still are – widely used in cardiovascular research, 3D cell-based systems have the potential to add a layer of complexity and to generate cardiac-like structures which faithfully recapitulate cellular interactions as those *in vivo*. Furthermore, 3D systems may also integrate scaffolds and biomaterials, mimicking the properties of the extracellular matrix present in the human heart. Thus, in the last years, 3D cell cultures are emerging as a new tool for both drug discovery and regenerative medicine applications. Indeed, the increased knowledge in the cardiovascular biology field boosted the development of 3D cellular systems, that progressively and faithfully reproduce the morphological and functional features of cardiac tissue, overcoming the maturation-related limitations of 2D cultures. A growing number of studies have indeed demonstrated that hPSC-CMs differentiated in 3D systems acquire structural, metabolic and functional properties similar to their adult counterparts (Fong et al., 2016; Correia et al., 2018). On this regard, it is worth mentioning the recent study from Ronaldson-Bouchard et al. (2018) in which the authors were able to obtain a level of maturation unprecedently seen in human iPSC-CMs through the combination of a fibrin 3D-hydrogel and physical conditioning with increased intensity (stretch and auxotonic contractions). Overall, several types of 3D cellular models have been developed in the last decade for their application in the cardiovascular field. Based on the presence of a supporting scaffold or not, these models can be broadly divided in two groups: (i) spheroids and organoids, which are formed by self-assembly (scaffold-free) and are able to recapitulate native tissue structure and functionality without the use of any exogenous support, and (ii) engineered cardiac tissue constructs which instead combine the use of different exogenous scaffolds with living cells and eventually electro-mechanical signals.

### Spheroids and Organoids

Spheroids and organoids both refer to 3D culture systems with a specialized architecture and cell organization that typically form through self-assembling processes. Currently, a precise and universal nomenclature that clearly distinguishes between these two models is still missing; thus, these terms are often used interchangeably.

#### Spheroids

Spheroids were first obtained in 1970 (Sutherland et al., 1971; Freedman et al., 2015; Takasato et al., 2015) to mimic the functional phenotype of human tumor cells and test their response to radiotherapy. Since then, several spheroid culture systems have been developed starting from different types of stem cells. This methodology presents several advantages with respect to the monolayer cell culture, particularly due to the spheroids’ cell heterogeneity: these culture systems, in fact, include diverse cell-type populations that consume different kinds of nutrients and secrete distinct metabolites and soluble signals. One crucial point to consider in spheroid aggregation is their size, namely the number of cells per spheroid. In fact, if the number of cells is too high, it will result detrimental for cellular viability due to a reduction in the oxygen supply at the center of the spheroid (Tan et al., 2017). Tan et al. (2017) have recently described the use of human iPSC-CMs in the formation of cardiac spheroids; interestingly, the authors demonstrated that use of electrically conductive silicon nanowires (e-SiNWs) ameliorates self-assembly of the CMs during the spheroid aggregation process. Furthermore, the resulting construct displayed improved cardiac functionality, probably due to an increased cell maturation mediated by the electrical stimulus (Tan et al., 2017). Recently, Mattapally et al. (2018) also employed human iPSC-CMs to produce spheroids, demonstrating that such spheroids fused in culture, generating structures characterized by a uniform distribution of cells and electro-mechanical coupling. Relevantly, they also showed that such structures, embedded in a fibrin patch and transplanted *in vivo*, were able to engraft the infarcted area in a mouse model of myocardial infarction (MI), with an engraftment rate exceeding 25%, significantly improving cardiac function (Mattapally et al., 2018). Cardiac spheroids could also be generated by combining CMs with other cell types that typically compose the human heart, such as cardiac fibroblasts (CFs), attempting to recreate the heart microenvironment (Polonchuk et al., 2017; Hoang et al., 2018; Yan et al., 2019). On this regard, Choi and coworkers generated homotypic and heterotypic spheroid-derived cardiac microtissues, containing either CMs or CFs or both cell types together (Kim T. Y. et al., 2018). The authors evaluated the electrophysiological properties of these tissue-like microstructures, focusing their investigation on the role played by CFs in cardiac action potential propagation. Results emerged from this study support the hypothesis of a central role played by the “supporting” cells in mimicking heart environment, indicating CF sodium currents as key players in action potential conduction in CF/CM heterotypic spheroids. Importantly, this study also reported a delayed action potential propagation in spheroids composed by disproportioned distribution of CFs, as it occurs in the infarcted myocardium, further sustaining the role of CF engraftment in the arrhythmogenic defects associated MI and the utility of 3D hetero-cellular models for studying cardiac diseases and for the development of regenerative strategies. Altogether, these studies support the idea of using 3D structures made of iPSC-derived CMs and other cardiac cells as heart surrogates for cardiac repair.

#### Organoids

An organoid is defined as an organ-like tissue exhibiting multiple cell types that self-organize to form a structure not unlike the organ *in vivo* and functional (Lancaster and Knoblich, 2014). As already mentioned above, there is not a clear distinction between organoids and spheroids, and in the past, the two terms were often used as synonyms. However, even if sharing several features, such as self-assembly properties, multicellular composition and some functional cues, organoids and spheroids are characterized by few distinctive traits. In fact, while the latter are basically cell aggregates without a defined tissue-like structure, organoids typically exhibit a higher order of self-assembly and do show, at least in some cases, a distinctive tissue-like cellular architecture. Therefore, over the last few years, the concept of organoids has changed and researchers usually refer to organoids to indicate a 3D tissue-like structure generated through self-assembly, while use of the term spheroid is limited to indicating a “premature” stage of what will then become an actual organoid.

The signaling pathways that control organoid formation and development were found to be the same as those occurring during the development of the organ itself. For this reason, growth factors and small molecules were used to manipulate different signaling pathways in order to increase cell survival, cell specification and organization of tissue-specific organoids. The first organoid was created in 2009 and was used to build intestinal tissue *in vitro*, through the establishment of a long-term culture system able to create the crypt–villus organization and physiology. The induction protocol relied on the use of a cocktail of small molecules required for the growth of the intestinal epithelium, such as Wnt proteins, epidermal growth factor and Noggin, which lead to sequential morphological changes and the formation of crypt domains (more than 40 per organoid) with a central lumen and an epithelium containing microvilli structures (Sato et al., 2009). Since then, many organoids have been developed to reproduce several organ-like structures including brain, lung and liver (Huch et al., 2013; Lancaster et al., 2013; Chen Y.-W. et al., 2017), so that in the recent years, the use of organoids has rapidly grown in several fields of medical research.

Despite these advances, very few studies have focused on the development of human cardiac organoids, and the obtained structures are still far from resembling the actual heart. This is mainly due to the structural and functional complexity of the heart, which is formed, in addition to CMs, by other cell types, such as CFs, vascular and immune cells, finely organized and embedded in the extracellular matrix to create its functional compartments. Thus, recapitulation of the 3D-multicellular structure and function of the developing myocardium through the use of human cardiac organoids remains a challenge. Given their multiple developmental potential, iPSCs represent a cell source of selection for the creation of cardiac organoids: manipulation of iPSCs is feasible and protocols are now available for specifically differentiating all the aforementioned heart-relevant cell types. It is indeed clear that to create a functional human cardiac organoid, the simultaneous presence of CMs, endothelial cells (ECs) and CFs is highly desirable. On this regard, Richards et al. (2017) have devised a developmental-driven scaffold-free fabrication strategy to assemble a functional vascularized cardiac organoid: their approach was inspired by the events that guide myocardial cell organization during heart development and was based on the self-assembly of human iPSC-CMs, -CFs and -ECs, that ultimately acquired a well-defined structure, exhibiting both biochemical and functional properties typical of the myocardium and being able to respond to drug treatments. Recently, Wimmer et al. (2019) also developed human organoids of blood vessels as models for studying diabetic vasculopathy. To this end, they induced PSCs (either iPSC or ESC) to commit toward a mesodermal lineage and then vascular specification using specific growth factors, that resulted in the generation of 3D blood vessel organoids possessing morphological and functional properties of the human microvasculature. Similarly, use of co-culture systems with iPSC-derived ECs, vascular cells and pericytes have been also employed by other researchers to recreate the vascular network (Kusuma et al., 2013; Orlova et al., 2014; Chan et al., 2015).

However, while the self-organization property of the organoids allows generation of a high level of tissue organization, one that cannot be reached by the current tissue engineering approaches with the only requirements of external cues and growth factors cocktails, this characteristic also represents the main weakness of this approach. In fact, the self-assembly process is not subjected to pre-defined extrinsic patterning instructions, but is instead a stochastic event, leading to heterogeneity in shape, size and cell composition (Brassard and Lutolf, 2019). In addition to this, the fabrication method limits the size of the organoids to the millimeter scale, thus resulting poorly applicable to regenerative medicine approaches. Recently, the generation of advanced engineered cardiac organoids that overcome some of the aforementioned limitations, such as sample heterogeneity, has been reported. This strategy, proposed by the James Hudson laboratory, implies the combination of bioengineering methods to the classical self-assembly protocol to obtain human cardiac organoids (hCOs) from PSC-CMs. More in detail, hCO constructs consisted of a mixture of differentiated PSC-CMs, collagen, sodium hydroxide and matrigel, which was then allowed to self-assemble on polydimethylsiloxane (PDMS) poles. The generated constructs were then used for investigating their regenerative capacity in response to injury (Eder et al., 2016) and for drug screening applications (Mills et al., 2019). However, although very advantageous for screening purposes, allowing to simultaneously evaluate multiple phenotypic parameters such as proliferation and side effects on cell functionality (i.e., contraction), this system is not ideal for developing cells for cell therapy. On this regard, conventional tissue engineering strategies are probably a more suitable option.

### Engineered Cardiac Tissue Constructs

Tissue engineering is an emerging and multidisciplinary field which aims at developing functional tissue substitutes for medical purposes, mainly replacement of damaged tissues/organs or drug screening applications. Tissue engineering involves the combinatorial use of cells, materials, biochemical and/or physical factors and engineering methodologies, and has thus far been applied to a large variety of human tissues (Vacanti and Langer, 1999). The bio-engineered scaffolds serve as structural support for cell seeding, while the addition of biochemical/physical factors determines distinct cell phenotypes and metabolic properties of the originated tissue. Clinical applications of tissue engineering approaches are highly relevant for repair of bone (Jones et al., 2002; Sepulveda et al., 2002), cartilage (Cao et al., 1997; Risbud et al., 2001), and pancreatic tissue (Niknamasl et al., 2014). In addition to these, given the limited regenerative capacity of the heart, tissue engineering approaches are also ideal for cardiovascular regenerative medicine applications. The main goal is to create a cardiac graft which can be implanted and restore the functionality of the myocardium without major side effects. To this aim, tissue engineering intends to recreate the microenvironment of the cardiac tissue, in terms of cell composition, stiffness, geometry, physical and electrical stimuli and, extracellular matrix.

The first engineered-based approaches simply implied the addition of cells to natural or synthetic biomaterials, that should mimic extracellular matrix providing mechanical support to the cells. Zimmermann et al. (2002) provided the first report on a 3D engineered heart tissue (EHT), developed using neonatal rat cardiac myocytes mixed with collagen I and matrix factors, added in a circular scaffold and subjected to phasic mechanical stretch. After that, many researchers applied similar methodologies using embryonic or neonatal/fetal rat cells embedded into polymer-based or extracellular matrix-like scaffolds, and showed the engraftment of these 3D-EHT constructs into rodent cardiac tissue (Li et al., 1999; Taylor, 2004; Ravichandran et al., 2013; Wendel et al., 2014). Many other cell types have been also used for generating 3D-EHT, that include adult and neonatal cardiomyocytes (Soonpaa et al., 1994; Li et al., 2000), bone marrow-derived stem cells (i.e., mesenchymal, endothelial, and hematopoietic stem/progenitor cells) (Janssens et al., 2006), cardiac stem cells (Barile et al., 2007), smooth muscle cells (Planat-Benard et al., 2004; Rehman et al., 2004), and skeletal myoblasts (Murry et al., 1996). In addition to these, in the last years, the use of CMs differentiated from PSCs exponentially increased, mainly because of their wide developmental potential and the rapid spread of iPSC technology in research laboratories worldwide. On this regard, Stevens et al. (2009) described, for the first time, a strategy to create macroscopic scaffold-free patches of human cardiac tissue exclusively composed of human CMs derived from ESCs. These patches showed synchronous calcium activity, which is indicative of proper electromechanical coupling. In addition, these patches were able to contract spontaneously and, by modulating the number of cells used for fabrication, it was also possible to tightly control the patch size. However, in order to generate constructs with an actual translational value, cardiac tissue engineering must take into account the native characteristics of the cardiac tissue. Indeed, the optimal engineered support should be formed by cardiac cell populations included into an extracellular matrix similar, in molecular composition, to that of the native tissue. Also, proper vascularization is key for long-term cell survival and function *in vivo*. Therefore, such complexity is not easy to achieve due to several limitations, mainly represented by the response of the host to biomaterials used to build the supports and the limited sources of suitable human cells.

The choice of the biomaterial is indeed key for developing effective cardiac regenerative strategies; biomaterials can indeed have different impacts on survival, proliferation and differentiation of a specific cell type, and allow specific scaffold anchoring to generate a functional tissue. Biomaterials used in tissue engineering can be either natural (e.g., collagen, alginate, fibrin, and natural ECM) or synthetic [e.g., poly(ethylene glycol), poly(ε-caprolactone), and poly(glycerol sebacate)]; both types of polymers may represent an optimal choice for fabrication of tissue-like cardiac structures and, depending on the specific application, have been alternatively employed to develop engineered constructs for cardiac regenerative purposes (Kai et al., 2014; Wendel et al., 2014).

Collagen is probably the most important “biomechanical” protein of the human body and it represents one of the most relevant natural polymers used in the field of cardiac regeneration. Indeed, due to its abundance in the ECM, collagen provides an optimal support for cell attachment and growth, and several studies in rodents have demonstrated positive effects of collagen-based scaffolds (Gaballa et al., 2006; Miyagi et al., 2011; Van Vlierberghe et al., 2011). Researchers have used collagen as a scaffold into the damaged myocardium, and found that this material was able to induce the formation of new vessels, resulting into a beneficial effect against cardiac remodeling (Shi et al., 2011). Masumoto et al. (2016) recently reported the generation of engineered cardiac tissue using collagen I, matrigel and human iPSC-derived cells. In detail, the authors generated a construct characterized by three cellular compositions – CMs, ECs and vascular mural cells, all derived from iPSCs – and showed a functional maturation of these cells, when assembled in this collagen-based tissue-engineered structure (Masumoto et al., 2016). Furthermore, they also demonstrated myocardial replacement after injection *in vivo*. However, despite the reported beneficial effects, collagen is a stiff material and this characteristic can impede its mechanical integration into the heart. To overcome this problem, several strategies have been proposed and demonstrated the ability to tune its mechanical properties; among these, Deng et al. (2010) showed that addition of chitosan is sufficient to increase the compression modulus of collagen fibrils (Geng et al., 2018). Indeed, the compression modulus of the collagen increases when combined with chitosan or elastin, whereas its tensile modulus is reduced, conferring greater stability to the heart and minimizing the ventricular wall dilatation (Fakoya et al., 2018). On this regard, it is worth mentioning the very recent work from Lee et al. (2019), who developed the FRESH methodology to 3D-bioprint collagen with improved mechanical properties, allowing the fabrication of cardiac components of different sizes.

Alginate is another natural material suitable for tissue engineering applications. It is an anionic polymer obtained from brown seaweed and it has been employed for numerous biomedical applications. In fact, alginate possess several characteristics – such as biocompatibility and low toxicity - that made it an ideal candidate for its medical use (Lee and Mooney, 2012). Alginate hydrogels can be produced by various cross-linking methods and the resulting structure is similar to the ECM, so that a positive effect on cardiac functionality has been reported with its injection in animal models after induction of MI, probably through a mechanism of reinforcement of the thickness of the scar (Landa et al., 2008). Despite these positive effects, the lack of integration between alginate and CMs is a major drawback of this biomaterial, limiting its full application for cardiac regeneration (Geng et al., 2018). However, a more recent study has reported the generation of a multi-cellular engineered construct containing alginate and iPSC-CMs (Maiullari et al., 2018). In this work, Maiullari et al. (2018) encapsulated iPSC-CMs into a hydrogel containing alginate and synthetic polyethylene glycol (PEG)-fibrinogen followed by custom bio-printing, and demonstrated an improved integration of this engineered support within the host tissue. As for the collagen, the combined use of alginate with chitosan has shown beneficial effects in a rat model of MI, mitigating ventricular remodeling.

Another natural biomaterial with some applicability in regenerative therapy is fibrin, an insoluble protein formed from fibrinogen. Use of fibrin has been reported to improve cardiac function by reducing infarct size (Deng et al., 2015). However, fibrin poorly tolerates the mechanical stretch generated during the contraction of the cardiac muscle and, for this reason, is usually used in combination with other materials such as chitosan or collagen (Christman et al., 2004). Likewise, hyaluronic acid, a polysaccharide present in the ECM, is a good candidate for regenerative medicine applications and has been shown to be involved in angiogenesis and tissue repair. Indeed, hyaluronic acid has been largely utilized to develop scaffolds for tissue regeneration in different research areas and many clinical trials have already started to evaluate the regenerative power of hyaluronic acid-based materials for treatment of different kinds of tissue lesions as ulcers, osteo-articular and bone damages (Bonafè et al., 2014). With regards to the cardiac field, hyaluronic acid-based constructs have been used so far in pre-clinical models where they showed a positive effect on regeneration after MI (Yoon et al., 2009). In agreement with this, Abdalla et al. (2013) reported a recovery of rat heart functionality after injection of hyaluronic acid into the peri-infarct area in a sub-acute model of MI. However, this positive effect on regeneration was lost in a chronic model of MI, indicating that the injection timing of the therapeutic agents is also a major determinant to drive cardiac repair (Yoon et al., 2014).

The use of a decellularized extracellular matrix (dECM) directly isolated from tissues represents another option to build scaffolds for cardiac tissue engineering and has been applied in several conditions, from myocardial infarction to orthopedic injures (Shen et al., 2011; Perea-Gil et al., 2016; Hernandez et al., 2018; Hussey et al., 2018). dECM contains adhesion proteins, such as laminin or fibronectin, able to promote cell attachment, and retains biochemical cues and mechanical properties of the native tissue; these characteristics are critical for the subsequent recellularization of the scaffold. On this regard, it has been shown that porcine-derived myocardial dECM-based hydrogels are able to promote infiltration of cardiac progenitor and endothelial cells (Singelyn et al., 2012). In addition, Hernandez et al. (2018) used an analogous dECM-based system to deliver microRNA and extracellular vesicles. More recently, Tsui et al. (2019) proposed a new hybrid scaffold fabricated by combining porcine-derived dECM and reduced graphene oxide (rGO), demonstrating that it provides a valid microenvironment for both cellular and tissue development when used to create EHT constructs. The authors also showed that such dECM-rGO-based EHT structures improve cardiac contractility of hiPSC-CMs. Likewise, Goldfracht et al. (2019) demonstrated that hiPSC-CMs embedded into a chitosan-enhanced dECM hydrogel possess a more mature phenotype than their 2D-cultured counterparts and form EHT constructs with an anisotropic muscle structure, in which phenotypes of inherited forms of arrhythmias can be reproduced and drugs can be tested. In particular, in the context of dECM-based materials, the injectable forms have gained much interest in the field, due to their ease of delivery to the host tissue (Hernandez et al., 2020). The first clinical trial was recently reported, testing an injectable ECM-hydrogel derived from decellularized porcine myocardium, called VentriGel (Traverse et al., 2019). The results obtained so far in a cohort of post-MI patients with severe left ventricular dysfunction support the safety of the *trans*-endocardial injection of the hydrogel.

A valid option to the use of natural biomaterials is given by synthetic polymers. The major advantage of synthetic polymers lies in the possibility to synthetize materials with the optimal physico-chemical and mechanical properties resembling at best those of the tissue of interest; on the other hand, low biocompatibility and potential toxicity may represent a limitation and must be excluded prior to their application *in vivo*. With respect to the cardiovascular field, the most popular synthetic polymers are represented by poly(ethylene glycol), poly(ε-caprolactone), poly(propylene), poly(vinyl alcohol), poly(*N*-isopropylacrylamide), poly(glycerol sebacate), poly(ester urethane)s (Chen et al., 2010; Martins et al., 2014; Zhou et al., 2014; Moorthi et al., 2017; Denis et al., 2019). The authors can refer to the following reviews for additional details (Arnal-Pastor et al., 2013; Cui et al., 2016). In addition to these, several synthetic materials with electroconductive properties have been also developed to improve cardiac conduction and maintain the electrical integrity of the heart. Electroconductive materials used for this purpose include: nanoparticles (Zhu et al., 2017), nanowires (Wickham et al., 2016), carbon nanotubes (Shin et al., 2014; Roshanbinfar et al., 2018), and polymers (Kaur et al., 2015; Mohammadi et al., 2017). Electroconductive polymers, such as polyaniline (PANi) (Hsiao et al., 2013) and polypyrrole (PPy) (Wang et al., 2016) could be incorporated into 3D-printed scaffolds and have been used for developing engineered tissue constructs, also in the cardiac field. Among the others, PANi and PPy are widely used in the context of cardiac repair, due to their environmental stability and electrical properties (Razak et al., 2015). Different studies have shown that application of these polymers leads to an increase of connexin 43, the most abundant isoform of gap junctions in CMs, critical for impulse propagation within the heart (Nishizawa et al., 2007; Wang et al., 2016). However, insolubility of these polymers in water represent a major limitation for their full application (Wang et al., 2017). Indeed, they are preferentially used in “classical” cell-seeding approaches, in which cells are seeded onto a pre-fabricated scaffold, rather than being simultaneously encapsulated with cells into inks for 3D bioprinting.

A valid alternative option is represented by polythiophene polymers, that are biocompatible and highly soluble in water (Hempel et al., 2017; Sinha et al., 2017). In particular, poly(3,4-ethy lenedioxythiophene):polystyrene sulfonate (PEDOT:PSS) is a promising candidate, able to support mouse stem cell-derived CMs attachment (Jiang et al., 2017). Using these polymers, Roshanbinfar et al. (2018) developed an electroconductive bio-hybrid hydrogel, which is able to enhance maturation and physiological properties of engineered cardiac tissues. In detail, the authors incorporated PEDOT:PSS in a collagen–alginate hydrogel creating an electroconductive construct structurally similar to the native ECM. In addition, hiPSC-CMs seeded on top of this polymer showed increased speed of contraction, high contraction amplitude, sarcomeric lengths more similar to adult CMs and synchronous rhythmic beating. More recently, the same research group developed a new construct able to mimic the native cardiac ECM in both structure and electrical activity (Roshanbinfar et al., 2020). For this purpose, they generated electrospun fiber mats using collagen, HA and PANi as synthetic polymer in different concentration. They used hiPSC-CMs to check the biocompatibility and the functionality of these supports, reporting an improved electrical coupling and contraction of the cells. Thus, within the last years, many biomaterials have emerged as good candidates for cardiac tissue engineering to fill the existing gap for their application to cardiac regeneration and treatment of MI. Notably, injectable materials, dECM and cardiac patches have been shown to be able to increase cell retention, improving cell survival and coupling with the host (Hastings et al., 2015; Cambria et al., 2017; Park et al., 2019). A summary of these biomaterials is provided in the Table 1, together with their *pro* and *cons*.

**TABLE 1:** Biomaterials used for cardiac tissue engineering.

Materials	Advantages	Disadvantages	References
**Natural materials** • Collagen	Biocompatibility, biodegradability and high cell proliferation rate	Stiffness makes difficult the integration within the heart	Shi et al., 2011; Masumoto et al., 2016; Geng et al., 2018
• Alginate	Biocompatibility and gelation capacity	Lack of integration with CMs	Landa et al., 2008; Geng et al., 2018; Maiullari et al., 2018
• Fibrin	Biocompatibility, high cell adhesion	Poor resistance to mechanical stretch	Christman et al., 2004; Deng et al., 2015
• Hyaluronic acid	Biocompatibility, high cell proliferation rate	Low mechanical properties	Abdalla et al., 2013; Bonafè et al., 2014
• Decellularized extracellular matrices (dECM)	Biocompatibility and promotion of cell attachment	Batch-to-batch variability	Singelyn et al., 2012; Hernandez et al., 2018; Spang and Christman, 2018; Goldfracht et al., 2019; Traverse et al., 2019; Tsui et al., 2019
**Synthetic polymers** • poly(ethylene glycol) poly(ε-caprolactone), poly(propylene) poly(vinyl alcohol), poly(*N*-isopropylacrylamide), poly(glycerol sebacate), poly(ester urethane)s	Possibility to customize material’s properties	Low biocompatibility. Poor cell adhesion and proliferation.	Martins et al., 2014; Zhou et al., 2014; Moorthi et al., 2017
• Electroconductive polyaniline (PANi) and polypyrrole (PPy) polymers	Possibility to customize material’s properties. Environmental stability and electrical properties	Low biocompatibility and insoluble in water	Hsiao et al., 2013; Razak et al., 2015; Wang et al., 2016
• Electroconductive Polythiophene polymers	Possibility to customize material’s properties, soluble in water	Limited processability	Richardson-Burns et al., 2007; Kaur et al., 2015
• Electroconductive PEDOT:PSS polymers	Possibility to customize material’s properties, support cell attachment, thermal, electrical and chemical stability	Limited processability, need to be combined with other supporting materials	Hempel et al., 2017; Jiang et al., 2017; Roshanbinfar et al., 2018, 2019

Another determinant factor to be considered when assembling an engineered tissue construct regards the scaffolds’ assembly: many different methodologies have been developed to optimize their composition, structure and biocompatibility. Use of self-assembled monolayers (SAM) of different polymers by hydrogen bonds and hydrophobic or electrostatic interactions represents one of the proposed approaches to produce natural biomaterials (Mason and Shimanovich, 2018). This technique is particularly suitable to mimic the extracellular matrix (ECM) structure, which is important for cell adhesion and survival (Oh et al., 2003). Both synthetic and natural polymers may also be assembled in scaffolds by electrospinning, which is another, and widely used, methodology to produce nanofibers (Kitsara et al., 2017). Lastly, thermally induced phase-separation (TIPS) also represents a robust methodology for 3D scaffold production. This technique entails few crucial steps (polymer dissolution, a phase of separation, polymer gelation, extraction of the solvent from the gel and freezing after drying) that result in the production of a nanofibrous foam, very similar in size to the natural collagen present in the ECM (Nguyen et al., 2019). In this context, 3D-bioprinting is also emerging as another valuable technology to produce improved scaffolds that may include cells, matrices and bioactive molecules for cell growth. 3D-bioprinting refers to the generation of physical 3D platforms, made by deposition of materials with a progressive layered approach, which is regulated by custom digital designs (Ong et al., 2018). Bio-printed structures could be generated with different technologies, such as inkjet bioprinting, laser-assisted or micro-extrusion bioprinting; the choice among these strictly depends on the desired outcome (Murphy and Atala, 2014; Vukicevic et al., 2017). During the last decade, 3D-bioprinting has provided us with the possibility to create organized tissue-like structures, that contain living cells, including CMs (Gaetani et al., 2012). As a matter of fact, Gaetani et al. (2012) were able to generate a cardiac tissue *in vitro* by combining bioprinting techniques and human cardiac progenitor cells (hCPCs), demonstrating the applicability of bioprinting in this area of research. Specifically, they demonstrated that hCPCs can be printed in a scaffold containing alginate without affecting cell proliferation and viability; additionally they showed an improvement in the cardiac commitment of the “printed” cells, that exhibited a significant increase of the expression of both, early (i.e., Nkx2.5, GATA-4, and Mef2c) and late (i.e., cardiac troponin T - TnT) cardiac-specific markers (Gaetani et al., 2012). More recently, Noor et al. (2019) made an important step forward in the field by developing a 3D printing technique that employs patient-derived hydrogels as bioinks and iPSC-derived CMs and ECs: by specific combination of these acellular/cellular components, they reported the generation of thick and vascularized cardiac patches that match the immune-cellular properties of a specific patient. More in detail, the authors started from a biopsy of omental tissue from which they isolated cells and ECM: cells were employed to generate CMs and ECs through reprogramming and subsequent differentiation, while ECM was processed to a form a personalized hydrogel, used to blend the cell-specific bioink formulations. The cardiac patches described in this work are the result of the printing of these two distinct bioinks into superimposed layers, each containing parenchymal tissue (CMs) and blood vessel forming cells (ECs). In addition to vascularized cardiac patches, the technique was also applied to fabricate small-scale cellularized perfusable human hearts with a natural anatomical architecture and mechanical properties. Likewise, later in the same year, Lee et al. (2019) reported an improved methodology for collagen 3D-bioprinting, using freeform reversible embedding of suspended hydrogels (FRESH). Using this approach, the authors were able to engineer different components of the human heart, ranging from small capillaries, to the valves, and the whole organ (Lee et al., 2019). By finely tuning the collagen gelation process, it was possible to control the morphology and the size of the printed filaments, increasing the resolution power of the bioprinting process. Importantly, the authors showed that the cardiac components obtained using their improved FRESH-based 3D bioprinting accurately reproduce structural, mechanical, and biological properties of the native tissue.

However, regardless of the employed strategy, generation of tissue-like engineered structures suitable for regenerative medicine applications needs further considerations, specifically in relation to some aspects that may be critical for their efficacy and potential undesired effects *in vivo*. As already mentioned above, the choice of the biomaterial utilized for developing engineered cardiac constructs is one crucial aspect, since this material will constitute the support for the cells, and should guarantee the optimal conditions for their growth, differentiation and survival. Another important parameter to be considered is represented by the “structure” (or “shape”) of the engineered supports, which provide the geometrical patterned for cell organization and for the establishment of cell-cell communication and 3D-microenvironment. Although there is no clear distinction between the different types of engineered-based constructs, based on their geometry and the methodology used for cell assembly, we can mainly identify cell sheets, cardiac patches and actual 3D-engineered cardiac tissue models.

#### Cell Sheets and Cardiac Patches

One of the first examples of a tissue-like structure obtained through engineering-based methodologies is represented by cell sheets. Cell sheets are generated by detachment of monolayers of cells, grown at confluency, from the culture surface. Functional tissues can be then fabricated by layering the recovered cell sheets without the need of any scaffold or complicated manipulation. Cell sheets are also used to produce engineered cardiac patches containing aligned CMs. Compared to cell sheets, cardiac patches are thicker engineered supports, which recreate a functioning piece of “heart tissue” and can be used to replace the damaged area of a patient’s heart, to maintain its contractile properties.

However, also in this case, it is difficult to establish a clear distinction between the two typologies of constructs. Compared to injection of isolated cells, CM-formed cell sheets showed enhanced cell vitality, heart-like electrical and histological properties with contractile ability *in vitro*; when transplanted *in vivo*, they were functional and electrically coupled with the recipient myocardium (Sawa et al., 2012).

In terms of technology, detachment of intact cell sheets from the culture surface may be achieved using different approaches. On this regard, Sawa et al. (2012) proposed a novel protocol based on poly(*N*-isopropylacrylamide) (PIPAAm), which is temperature-responsive polymer. Under normal culture conditions (37°C), the surface of the dishes is relatively hydrophobic and cells efficiently attach and proliferate. In turn, when temperature decreases to about 32°C (the polymer’s lower critical solution temperature), the polymer becomes hydrophilic and a hydration layer forms between the surface of dishes and the cells, leading to their detachment from the culturing surface as an intact sheet, with no need of enzymatic treatments. Using the same approach, Masumoto et al. (2016) reported the use of cell sheets, formed by different cardiac cells generated through human iPSC differentiation *in vivo*, and demonstrated their ability to drive functional recovery after MI. More recently, Ishigami et al. (2018) demonstrated functional restoration of the failing myocardium also in a porcine model using human iPSC-derived cardiac sheets. In their work, the authors induced differentiation of iPSC into CMs, vascular cells and vascular mural cells and generated the cardiac sheets using 10 cm-sized temperature-responsive culture dishes. When transplanted *in vivo*, the generated cardiac sheets led to a significant reduction of the fibrotic area and an increase of the capillarity density in the border zone. However, the cell sheet layering methods still present some limitations mainly related to the number of cell layers that can be superimposed before incurring the decrease in oxygen and nutrients supply to the cells and thus leading to the necrosis of the transplanted patch after its implantation. On this regard, the recent work from Noor et al. (2019), already discussed above, gave a substantial contribution to the field, overcoming part of these limitations. By using 3D-printing techniques and iPSC-derived cardiac and endothelial cells encapsulated into tissue-derived hydrogels, they fabricated vascularized thick (2–7 mm) cardiac patches, that exhibit high cell viability and contractile activity. Future long-term *in vitro* studies and *in vivo* experiments will certainly establish the therapeutic potential of such cardiac constructs.

#### 3D-Engineered Cardiac Tissue Models

Engineered cardiac tissue models have recently emerged as promising approaches to repair damaged cardiac tissue as well as suitable platforms for drug/toxicity testing and disease modeling (MacNeil, 2007). Through the use of the natural and/or synthetic biomaterials mentioned above, it is possible to generate implantable supports able to mimic the architecture and composition of the heart ECM, that are necessary to optimize cell growth, differentiation and survival *in vivo*. In fact, engineered cardiac tissue models are generated with the aim of recreating the heart environment, and to provide the proper electrical and mechanical support to the cell for their delivery into the heart. Eschenhagen et al. (1997) were the first to develop a method to culture chick cardiac CMs in 3D structures using a collagen matrix. The authors showed the possibility to measure the isometric force of contraction generated by CM-populated matrices, later referred as engineered heart tissue (EHT). In addition, this matrix was suitable for genetic manipulation, showing an efficient transduction rate to adenoviral-mediated gene transfer of the populating CMs. Zimmermann et al. (2000) reported the development of EHTs using rat CMs and 2 years later, they provided an improved version of this technique creating new EHT geometry (Zimmermann et al., 2002). Thus, such engineered cardiac tissues were first used to deliver CMs to the infarcted area after MI in preclinical animal models. In the first attempt, despite an initial improvement in cell survival, the engineered structures were unable to ameliorate cardiac function *in vivo* (Li et al., 1999). However, different results were subsequently obtained using cardiac engineered tissues with similar composition, that were instead associated to an increase in cell survival and ameliorated cardiac function (Leor et al., 2000; Akhyari et al., 2002; Eschenhagen et al., 2002; Kofidis et al., 2002). To give some examples, Eschenhagen et al. (2002) used collagen-based EHT to restore function of the damaged myocardium, demonstrating long-term survival and contraction of these structures *in vivo* (over 8 weeks after implantation). Similar results were obtained by Dar et al. (2002) using alginate-based scaffolds.

The improvement of protocols for differentiation of PSCs toward the cardiac lineage made the production of large amounts of human CMs feasible, bursting the development of human heart surrogates for disease modeling, drug testing and autologous cell therapy applications. On this regard, embedding CMs derived from PSCs into engineered structures has been shown to positively impact cell maturation to different extents, depending on the specific characteristics of the generated 3D-constructs. In fact, even if improved over time, use of both human iPSCs and ESCs still deals with an important issue of cell maturation following cardiac induction *in vitro*. One of the first evidence supporting the maturation of CMs in 3D-engineered constructs is given by the study from Schaaf et al. (2011), who developed a EHT using CMs derived from human ESCs and showed that, when included into the fibrin-based 3D-constructs, despite an immature electrophysiological profile, these ESC-derived CMs displayed a more organized sarcomeric structure compared to those cultured in 2D under conventional conditions. In the same year, the group of Charles E. Murry also developed collagen-based bio-engineered human cardiac tissue constructs using CMs derived from both ESCs and iPSCs, demonstrating an improvement in their alignment and proliferation (Tulloch et al., 2011). They also found that these engineered constructs were able to engraft into the heart establishing connections with the host vasculature. Similarly, Mannhardt et al. (2016) investigated morphology and function of EHTs built using human iPSC-CMs, and tested their suitability for drug screening. In detail, these EHT constructs were anchored to two flexible silicone supports that generated a preload against which the EHTs perform auxotonic contractile work. This process led to the formation of muscle bundles in which CMs showed a better alignment and an improved sarcomeric organization. Thus, altogether these studies strongly indicate that EHT constructs are excellent supports for maturation of CMs derived from human PSCs, since they provide a physiological-like environment and stimuli to cells.

On this regard, the recent work from Ronaldson-Bouchard et al. (2018) gave a substantial contribution to the field. In their work, the authors showed that exposure of early-stage iPSC-CMs to myocardium-like mechanical forces and electrical stimuli at increasing intensity (from 2 to 6 Hz in 4 weeks) enables cells to reach unprecedent levels of maturation. In fact, the authors reported an upregulation of late-stage cardiac-specific markers, the length of the sarcomeres (up to 2.2 μm), the presence of intercalated disks and T-tubules, the high density of mitochondria, the switch to oxidative metabolism and the establishment of a more mature functional phenotype. Indeed, even if still inferior to the adult myocardium, the generated cardiac constructs showed more mature electrophysiological characteristics and calcium handling, increased conduction velocity, complete responsiveness to β-adrenergic stimulation and a positive force-frequency relationship (FFR), which is a stringent indicator of maturation never seen before in other EHT models (Ronaldson-Bouchard et al., 2018).

Overall, thanks to these improved characteristics, EHTs may be used as a model platform for the *in vitro* study of human cardiac functions, in terms of the force, pacemaking activity, contractile properties and electrophysiological parameters (Hansen et al., 2010; Mannhardt et al., 2016), in addition to representing a promising resource for future cardiac regenerative therapies. With respect to cardiac regeneration, preclinical studies conducted in a guinea pig model of MI showed an improvement of the left ventricle function after transplantation of iPSC-derived EHTs; in some cases these 3D-EHT constructs were also shown to be coupled to the host cardiac tissue (Weinberger et al., 2016). Recently, another study by the same research team, also assessed the issue of the increased risk of cardiac arrhythmia secondary to EHT implantation (Pecha et al., 2019). This analysis was performed through the implant of telemetric devices following EHT transplant and the monitoring of the electric activity of the heart for 28 days: results demonstrated that in this case, the incidence of ventricular arrhythmias was not affected by EHT transplant. However, other reports showed discordant results, demonstrating a link between transplantation of iPSC-derived CMs and induction of transient abnormal beating, potentially leading to malignant ventricular arrhythmia (Chong et al., 2014; Shiba et al., 2016). Furthermore, despite the encouraging results obtained from using cardiac-engineered tissues for cell transplantation *in vivo*, other studies reported a limited effect of cardiac patches, dampening the initial enthusiasm on their potential translation to the clinic in the short time. To give an example, in a recent study Sugiura et al. (2016) employed tissue-engineered biodegradable patches [made of polyglycolic acid poly(l-lactic-co-ε-caprolactone) copolymer] seeded with iPSC-CMs and tested their applicability *in vivo*. Their results showed that, at 4 weeks post-implantation, no iPSC-CMs remained in the patch (Sugiura et al., 2016), warning about the need of punctual experimentations that take into account all the potential variables (biomaterial, type of cells, degree of cardiac injury) before application of such tissue substitutes in patients. In this context, Park et al. (2019) recently demonstrated that iPSC-CMs and human mesenchymal stem cell-loaded patches are able to increase cardiac regeneration in a rat model of MI, improving cell retention and engraftment within the myocardium. The presence of human mesenchymal stem cells into the patch is likely providing a favorable microenvironment for vascular regeneration (Park et al., 2019). Very recently, similar work from the group of Charles E. Murry contributed to the field adding an additional layer of complexity to such tissue engineered constructs, and highlighting the importance of vascularization. Indeed, insufficient vascularization is one of the major problem of application of EHT for cardiovascular regeneration (Redd et al., 2019). In this work the authors combined stem cell methodologies and advanced tissue engineering to develop an engineered perfusable microvasculature system; specifically, they assembled ECs generated from ESCs into patterned micro-channels and a collagen matrix and demonstrated that, in these engineered constructs, ECs were able to form *de novo* vasculature through neo-angiogenesis and vascular remodeling processes. Furthermore, when transplanted *in vivo* in a rat model of MI, such perfusable microvascular graft integrated with the host coronary with a greater extent compared to non-perfusable constructs. Interestingly, when CMs were also embedded into the grafts, these were shown to support their survival after implantation, further supporting the importance of vascularization in the design of engineered cardiac tissue constructs for regenerative purposes. Altogether these studies showed that cardiac engineered constructs enhance intercellular organization and crosstalk of iPSC-CMs, improving their maturation *in vitro*, and ameliorate cardiac performance after the transplantation *in vivo*, thus providing encouraging evidences for their future use to achieve cardiac regeneration in humans.

## Transdifferentiation of Non-Contractile Cells: an Alternative Approach to Heart Regeneration

After myocardial infarction, the heart undergoes pathological remodeling, in order to preserve its function and the structural integrity of the cardiac muscle. During this process, necrotic cardiac tissue is replaced by non-CM components, mostly fibroblasts, that ultimately form fibrotic scar. The recent evidence that it is possible to directly induce the conversion of non-cardiomyocyte cells into CMs have emerged as a promising area to achieve *in situ* cardiac regeneration, by reducing the extent of the cardiac fibrotic scar (Talman and Ruskoaho, 2016). The first study dates back to 2010 and showed the direct reprogramming of postnatal cardiac or dermal murine fibroblasts into CM-like cells through the supply of a combination of three developmental transcription factors (i.e., Gata4, Mef2c, and Tbx5) (Ieda et al., 2010). In the same study, the authors showed the successful reprogramming into CMs when fibroblasts were transplanted *in vivo*. Subsequent studies further supported this evidence, showing the possibility to redirect the fate of cardiac fibroblasts (CFs) toward CMs *in vivo* through different strategies (Nam et al., 2013b; Doppler et al., 2015; Batty et al., 2016; Chen Y. et al., 2017).

Cardiac fibroblasts are abundantly represented in the adult cardiac tissue, where they play a crucial role both in physiological and pathological conditions: they are indeed crucial to maintain the normal cardiac function and are actively involved in cardiac remodeling leading to fibrosis and cardiac dysfunction (Batty et al., 2016). Local administration of the three cardiac transcription factors Gata4, Mef2c, and Tbx5 has been demonstrated to be sufficient to switch murine CFs toward a CM-like phenotype *in vivo*, and this was associated with a reduction of the infarct size and a modest mitigation of cardiac dysfunction after MI (Qian et al., 2012; Chen Y. et al., 2017). Along the same line, Fu et al. (2015) reported the induction of beating CMs from mouse CFs only by using a chemical cocktail, made of the GSK3β inhibitor CHIR99021, the inhibitor of the TGF-β type 1 receptor RepSox, Forskolin, valproic acid (VPA), the monoamine oxidase inhibitor Parnate, the analog of retinoic acid TTNPB, and the histone methylation inhibitor DZnep (3- deazaneplanocin A). These chemically induced cardiomyocyte-like cells (CiCMs) expressed CM-specific markers and exhibited evidence of sarcomeric organization (Fu et al., 2015).

Achievement of the simultaneous expression of all reprogramming factors in CFs is crucial for determining the efficiency of the reprogramming process. In fact, the reprogrammed fate is acquired within the first 48 h after *Gata4*, *Mef2c*, and *Tbx5* administration, and represents a critical step to be improved in order to overcome the extremely low efficiency initially reported for CF/CM transdifferentiation (Bektik and Fu, 2019; Stone et al., 2019; Zhang et al., 2019). On this regard, several methodologies have been developed in the recent years to enhance the efficiency of the “classical” transcription factor-based cardiac reprogramming protocol, including epigenetic modulation and treatments with inhibitors or cytokines (Nam et al., 2013a; Chen Y. et al., 2017). Addis et al. (2013) described an approach to optimize the direct conversion of fibroblast to CMs, using a reporter system in which the calcium indicator GCaMP was driven by the CM-specific Troponin T promoter. In addition to Gata4, Mef2c, and Tbx5, the authors also administered Hand2 and Nkx2.5, showing that this new transcription factor combination was 50-fold more efficient than the classical three cardiac transcription factor protocol and was able to induce CMs expressing specific markers and with robust calcium oscillation and spontaneous beating, which persisted after the subsequent inactivation of the reprogramming factors.

Based on this evidence, the possibility to achieve direct reprogramming of CFs into CMs *in vivo* holds great promise for restoring cardiac function. Diverse strategies have been already proposed, exhibiting a certain rate of success. Among the others, one interesting strategy for directing reprogramming of CFs into CMs relies on the use of microRNAs (miRNAs) (Jayawardena et al., 2012). Synthesized miRNA could be easily administered to cells of interest by lipid-based transfection, showing a low toxicity for the host (Elmén et al., 2008). Jayawardena et al. (2012) demonstrated that *in vitro* mouse fibroblasts could be directly reprogrammed to a cardiomyocyte-like phenotype using a specific cocktail of miRNAs (miRNAs 1, 133, 208, 499) and JAK inhibition. The “newly” generated CMs showed sarcomere organization and expressed cardiomyocyte-specific genes. At the functional level, the authors reported calcium transient and mechanical contraction (Jayawardena et al., 2012). Likewise, Muraoka et al. (2014) found that miR-133 is able to potentiate cardiac reprogramming by directly repressing Snai1 (master regulator epithelial-mesenchymal transition) and silencing the fibroblast signature. In the same way, Christoforou et al. (2017) described the transdifferentiation of human CFs toward CMs via the delivery of miRNAs miR-1 and miR-133a and the induction of the expression of Gata4, Tbx5, Mef2C, Myocd, and Nkx2.5. However, although the initial excitement on the potential use of direct cell reprogramming to achieve cardiac regeneration, the approach is still rather immature and not sufficiently tested for its translation into the clinic. Indeed, cardiac reprogramming efficiency is still very low (about 20% of cells expressing cardiac markers, corresponding to 1–2% of cells with recordable action potential and functional) and give rise to cardiac cells with an immature phenotype.

In addition to that, we should consider that cardiac reprogramming of human cells is more difficult – and therefore less efficient – compared to the murine counterpart. Wada et al. (2013) reported that the addition of Myocd and Mesp1 to three classical factors (Gata4, Mef2c, and Tbx5) could increase the switch of human cardiac and dermal fibroblasts into CMs. Similarly, Fu et al. (2013) reported that addition of *ESRRG* and *MESP1* genes was able to increase cardiac reprogramming from human cells, and to generate CMs showing sarcomere formation and cardiac-specific gene expression. Despite the low efficiency of the current protocols and the need of further studies to develop new and optimized direct reprogramming strategies, these reports are encouraging about the possibility to apply cardiac reprogramming to regenerative medicine in the future.

## Conclusion and Future Directions

**FIGURE 3:**
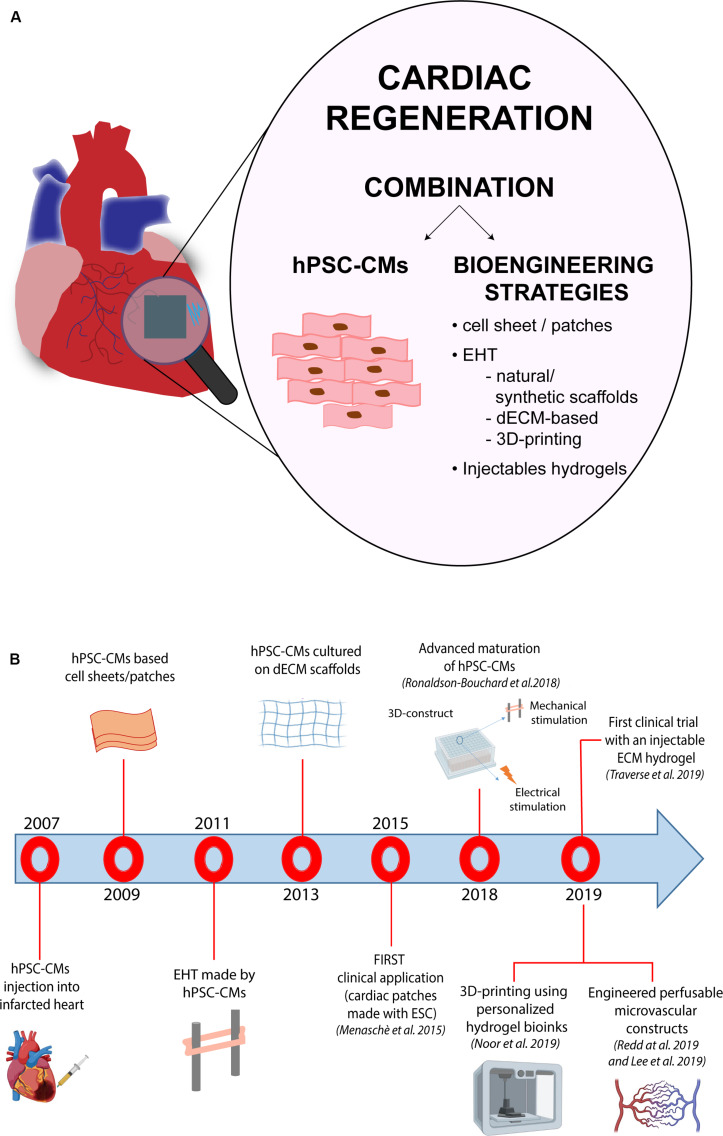
hPSC-based bioengineering strategies for cardiac regeneration. The panel **(A)** shows a schematic representation of the different hPSC-based methodologies used for regenerative purposes in the cardiac field. The panel **(B)** provides a time line that summarizes the key milestones reached in the field, starting from the simple injection of hPSC-CMs into the heart to the development of tissue-like structures with enhanced hPSC-CM maturation, more complex perfusable and personalized constructs and injectable hydrogels.

Although different types of stem cells have been proposed as potential candidates for cardiac regenerative medicine, there is no consensus about which represents the best choice. Given the complexity of cardiac tissue, which is composed by diverse cell types, human PSCs – mostly iPSCs – are certainly among the most relevant sources. In particular, CMs differentiated from iPSC may represent a realistic option for the regeneration of the injured heart. During the last few decades, researchers have made substantial progress in generating iPSC-CMs which have led to a general better understanding the biology of these cells and, to the definition of highly efficient differentiation protocols. A significant number of different cell therapy strategies that employ iPSC-CMs have been proposed to restore cardiac function, ranging from injection of cells alone, to their combination with ECM-like biomaterials, and to more complicated engineered-based strategies, which aim to faithfully recreate the myocardium. The majority of these approaches have shown – at least to some extent – success in mimicking heart cells and improving cardiac function, providing new hope for the replacement of CMs after the irreversible loss of heart tissue occurring during myocardial infarction (Figure 3). Notably, as mentioned before, Menasché et al. (2015) reported the first clinical application of cardiac patches composed of human ESC-derived cardiac progenitor cells in a patient suffering from severe ischemic left ventricle dysfunction, demonstrating the possibility to use a engineered-based approaches for cardiac repair.

More recently, iPSC-based therapies are taking over ESCs, offering a solution that overcomes ethical issues related to the use of embryo-derived material and offering a source of autologous cells for transplantation. Few clinical studies using iPSC-derived cells have already started to treat patients suffering from different diseases, such as neovascular age-related macular degeneration and Parkinson disease (Barker et al., 2017; Mandai et al., 2017). Some progress in this regard is being made also in the cardiovascular field: in May 2018, Japan’s government approved the transplantation of cardiac sheets made of CMs derived from human iPSCs to treat three patients suffering from heart disease (Cyranoski, 2018). Regardless of the questions arising with respect to this decision and whether or not it was too early to jump into clinics for iPSCs in the heart, results from this clinical trial (not available yet) will surely be crucial to set the stage for this kind of treatment in the cardiovascular field.

In this scenario, tissue engineering has emerged in the last years as a new field of research applied to cardiac tissue repair for developing improved strategies to treat cardiac dysfunctions after injury. Advantages of tissue-engineering based methodologies rely on the possibility to provide CMs with the biological environment mimicking that of the heart tissue: tissue engineering allows the creation of suitable three-dimensional matrices resembling the physico-chemical properties of the myocardium in which cells are maintained viable and functional, and therefore with a higher chance to positively act on cardiac regeneration (Table 2).

**TABLE 2:** Overview of iPSC-based 3D cell culture approaches.

Type	Bio-functional properties	Advantage	Disadvantage	References
**Spheroids**	Self-assembling multicellular aggregates.	Scaffold-free technology without the use of any exogenous support. Composition with different cell-type populations. Manipulation by pipetting and sedimentation. Size allows the miniaturized multi-well formats compatible with plate readers	Small size and heterogeneous composition of multicellular aggregates. No direct measurements of electrophysiological properties. Non-linear cell alignment. Number of cells: detrimental for the cellular viability.	Polonchuk et al., 2017; Tan et al., 2017; Correia et al., 2018; Hoang et al., 2018; Kim T. Y. et al., 2018; Mattapally et al., 2018; Yan et al., 2019
**Organoids**	Self-assembling organ-like tissue.	Composition with different cell-type populations. Mimics thicker tissues. High level of tissue organization. Size allows the miniaturized multi-well formats compatible with plate readers.	Stochastic events for self-assembly: heterogeneity in shape, size and cell composition. Size of multicellular aggregates. Number of cells: detrimental for the cellular viability.	Kusuma et al., 2013; Lancaster et al., 2013; Orlova et al., 2014; Chan et al., 2015; Eder et al., 2016; Chen Y.-W. et al., 2017; Richards et al., 2017; Mills et al., 2019; Wimmer et al., 2019
**Cell sheet cardiac patches**	Transplantable 3D-constructs.	Layering of CMs forming a tissue-like structure. Easy to produce and to manipulate. Precise control of cell sheet shape and structure.	Number of cell layers: possible decrease in oxygen and nutrients supply to cells; necrosis of the cells sheets after implantation.	Shimizu et al., 2006; Stevens et al., 2009; Gaetani et al., 2012; Vallée et al., 2012; Wendel et al., 2015; Masumoto et al., 2016; Ishigami et al., 2018; Noor et al., 2019; Park et al., 2019
**Engineered cardiac tissue models**	3D heart-like constructs with higher level of biological complexity.	Composition with different cell-type populations. Linear cells alignment. Improved maturation of hIPSC-CMs. Direct measurements of electrical activity of the cells.	Coupling with the host tissue. Vascularization. Biocompatibility of the materials.	Tulloch et al., 2011; Deng et al., 2015; Mannhardt et al., 2016; Masumoto et al., 2016; Sugiura et al., 2016; Weinberger et al., 2016; Hernandez et al., 2018; Maiullari et al., 2018; Ronaldson-Bouchard et al., 2018; Lee et al., 2019; Redd et al., 2019; Traverse et al., 2019; Tsui et al., 2019; Roshanbinfar et al., 2020

The progress made by tissue engineering technologies has been encouraging. Indeed, with currently available methodologies, it is feasible to create bio-supports able to incorporate the cells needed for the regeneration of cardiac tissue. However, besides the milestones reached thus far, significant improvements are still mandatory before we could consider their routine use in the clinical field for treatment of patients suffering from myocardial diseases. These improvements mainly regard scaffold production, through the careful analysis of biocompatibility and mechanical properties of the engineered constructs developed for the clinics. Also, further investigations on the interaction occurring between cells and the ECM and vascular integration with the host are highly desirable in order to further ameliorate tissue engineering-based supports, hopefully opening new doors for cardiac regeneration therapy via tissue engineering strategies. In this scenario, the possibility to directly convert fibroblast into CMs *in vivo* in the infarcted heart has emerged as potential option for cardiac regeneration. However, despite the promising results obtained by preclinical research, the approach is still rather immature for its clinical translation and further investigations are needed to develop effective and more efficient strategies to achieve cardiac regeneration *in vivo*.

## Author Contributions

MM performed the bibliographic search and wrote the manuscript. ED wrote and revised the manuscript and provided funding.

## Conflict of Interest

The authors declare that the research was conducted in the absence of any commercial or financial relationships that could be construed as a potential conflict of interest.
